# Are Malaysians Exercising? A Psychometric Analysis of Their Physical Activity Habits, Physical Literacy and Exercise Participation Rates among Adults with and without Disability

**DOI:** 10.3390/bs13070570

**Published:** 2023-07-10

**Authors:** Maziah Mat Rosly

**Affiliations:** Department of Physiology, Faculty of Medicine, Universiti Malaya, Kuala Lumpur 50603, Malaysia; maziahmr@um.edu.my

**Keywords:** questionnaire, physical disability, metabolic intensity, quantitative research, rural health

## Abstract

Background: Physical activity levels of adults worldwide have reported a rising trend in sedentarism. This study’s main objective is to analyze and understand the current tendency in this field and in physical literacy among Malaysian adults in order to improve physical performance. Methods: The sample from which the data were collected corresponded to 352 Malaysian participants (N = 176 non-disabled, N = 176 physical disability) using The Physical Activity Scale for Individuals with Physical Disabilities questionnaire. Results: Four factors were extracted, consisting of leisure activities, home maintenance, household chores, and career. The group with physical disability reported higher physical activity levels (14.30 MET h/day) compared to non-disabled (12.77 MET h/day), performing higher in leisure activities and light exercise. The compliance rate to health-beneficial exercise was 12.8% and was significantly higher among those with physical disability. Self-reported physical activity level correlated moderately well to overall MET performed *p* < 0.000, (r = 0.57). Only 2.8% of the respondents were aware of the recommendations outlined by health guidelines. Conclusion: The results indicated that the population surveyed was moderately active but had low compliance to exercise habits as recommended by international health guidelines. Non-participation in prescribed exercises was linked to higher education, urban dwellers, and higher income. The study also highlighted very low physical literacy among respondents in health recommended exercise guidelines.

## 1. Introduction

Adults, comprising youths and older adults aged 18–65 years old, need to exercise routinely in order to sustain an active lifestyle for continued health and cardio-metabolic benefits. Globally, international physical activity (PA) guidelines have recommended at least 75–150 min of moderate–vigorous exercise per week [1]. However, the World Health Organization (WHO) has repeatedly reported [2,3] concerning estimates of the physical activity levels (PALs) of the global population. The WHO’s most recent update [4] on PAL has reported consistent inactivity among 16.2–36.8% of the surveyed adult population. The vulnerable groups of high inactivity are more prominent in high income countries, older adult age groups, and female population [4]. Additionally, participation in moderate–vigorous intensity exercises in durations as recommended by various PA guidelines did not see much improvement over the last decade [4]. Even more worryingly, is that estimates have projected that this trend in PAL will not see a reduction in overall global inactivity by at least 15% come 2030 as initially targeted [5].

An important part of the initiative to improve PAL globally lies in the fundamental capability of individuals to establish physical pursuits in the form of physical literacy (PL), which is essential to their lifestyle [6,7]. PL [8] is the combination of four elements involving the affective, physical, cognitive, and behavioral aspects of physical activities. This involves the motivation and confidence (affective) to adopt PA as a lifestyle; the ability to develop physical competence (physical) in performing the necessary movement skills and patterns within a given setting; the knowledge and understanding (cognitive) of PA that allows them to understand the benefits of health, with the ability to appreciate appropriate safety concerns and essential qualities that allow movements; and finally engagement in physical activities for life (behavioral) that involves people taking action in engaging and sustaining in PA as a lifestyle [6,7,8]. Altogether, these aspects are defined by The International PL Association as “…the motivation, confidence, physical competence, knowledge, and understanding to value and take responsibility for engagement in physical activities for life” (IPLA, 2017, para. 1) [8]. 

Part of the range combined within the PL spectrum is the importance of education on health benefits, the cost of physical inactivity, and the types of PA appropriate for different individual’s needs [4,7]. These targeted areas of interest are important to relay to the general population, in order to increase their knowledge and understanding of PL and overall health [6,9]. In a survey conducted by Cai Lin et al. [10], education level was among the strongest predictor for PA status, and developed countries have shown that higher education was strongly correlated to higher PAL [11]. One of the most important aspects is the categorization of intensities when it comes to different types of exercise, sports, or PA [1,12]. In one of the surveys reported, participants were sometimes unsure of how to exercise when they had a disability and whether or not it was safe or feasible to exercise at a certain age or with a disability [13,14]. 

In order to improve global PAL, it is first very important to recognize the PA patterns and their associated factors affecting participation in PA or exercise for a specific adult population [15,16,17]. Understanding the reason behind a specific trend is essential in recognizing the problem within an iterative process and mapping them to a theoretical framework, in order to create more effective solutions. In this regard, the main objective of this study is to compare and contrast the descriptive patterns of PA performed and PL status according to two different populations, the non-disabled and individuals with physical disability. The study will also assess, characterize, and correlate overall PAL, activities of daily living (ADL), and exercise participation rates based on specific demographic factors. The findings of this cross-sectional study may help in understanding behavioral characteristics determining exercise participation rates and be able to provide direction for designing exercise intervention plans suitable for specific adult groups in future studies.

## 2. Materials and Methods

### 2.1. Questionnaire and Pilot Survey

The Physical Activity for Individuals with Physical Disabilities (PASIPD) questionnaire was used for this cross-sectional survey to identify and compare the PA spectrum and levels between adults with and without disability. The PASIPD questionnaire was designed for use among both the non-disabled and individuals with physical disabilities [18], using simple terms deemed appropriate for those with basic education of up to 12 years old and has been adapted to fit the Malaysian setting [16]. A pilot survey was conducted among 10 pre-test adults prior to distribution [13] in order to assess its fit for the target population. The assessment used a dichotomous scoring system (in the form of “clear” or “unclear” responses) for each individual item in the questionnaire, and revisions were made until at least 80% inter-rater agreement was achieved [19,20]. In this adaptation, item 12 had to be adjusted to include “taking care of a pet/animal” in order to fit individuals living alone in urban areas or farmers taking care of their farm animals, as this was a common practice among rural households in Malaysia. 

The PASIPD questionnaire uses the nomination metabolic equivalents of tasks (MET) hours over a 24 h period to describe the ADL performed by individuals over the past week. The format uses an objective measure to capture 12 different types of activities in frequencies (never, seldom: 1–2 d/week, sometimes: 3–4 d/week, or often: 5–7 d/week) and duration (<1 h, 1 but <2 h, 2–4 h, >4 h). The original paper for the PASIPD’s development and evaluation, by Washburn and team in 2002 [18], was intended for non-disabled individuals and individuals with physical disability. 

### 2.2. Participation Selection and Data Collection Procedure

Ethics application for this protocol was approved by the University of Malaya Research Ethics Committee, MREC ID NO: 2018223-6044 and UMREC-1892, which were approved on 23 September 2019 and 1 June 2022, respectively. The inclusion criteria encompassed all adults aged between 18–65 years old with coherent cognition and good comprehension in the national language, Bahasa Malaysia, either in verbal or written command. Respondents with a physical disability comprised those with spinal cord injury, cerebral palsy, spina bifida, amputations, stroke, chronic low back pain, or blindness, as recommended by Washburn et al. [18]. Individuals with a form of cognitive disease; intellectual disability; or underlying medical history requiring treatment that precludes them from taking part in moderate–vigorous exercise activities on a regular basis [21], particularly non-communicable diseases such as hypertension, hypercholesterolemia, type 2 diabetes, heart disease, or ischemic heart disease and chronic kidney diseases, were not eligible. The call for survey participation was disseminated to adults from all 13 states of Malaysia via email, flyers, posters, and brochures, which contained a brief information sheet regarding the study.

Following participant’s agreement to participate, distribution of the questionnaires was undertaken either physically or digitally via various forms of social communication [22], and informed written consent was taken at the start of the questionnaire. A research assistant helped individuals who may have required physical support during data collection. Demographic information such as age, type of disability, area, earning bracket, education level, relationship status, use of mobility aids, and perceived PAL [23] were also included. Additionally, the questionnaire inquired about participants’ knowledge regarding global recommendations for health improvements and their perceived level of PA, as well as the availability of exercise equipment they have at home and the frequency of its use.

### 2.3. Data Analysis

A total of 352 completed responses were collected using the recommended sample size of at least 10 responses per questionnaire item for reliability and comparative analyses, around N~50 for correlation coefficients and around N~300 for factor analysis [20,24]. All data were analyzed using Excel 2019 (Microsoft Corporation, Redmond, WA, USA) and SPSS version 25 (IBM, Armonk, NY, USA). The Shapiro–Wilk and Kolmogorov–Smirnov tests were used to assess for normality, and all data indicated non-normal distribution with *p* = 0.00 for both. Thus, non-parametric tests were used to analyze the subsequent data. Reliability analyses were conducted using Cronbach’s alpha based on the standardized items, in addition to intraclass correlations using a two-way mixed effects model.

MET h/day for total and individual PA between the two groups were compared using the Kolmogorov–Smirnov Z test, and effect sizes were estimated using Cliff’s delta for ordinal data and non-parametric tests. The absolute value for effect size estimation was considered small if around 0.147, medium if around 0.33, and large if around 0.474 [25].

Correlation tests using one-tailed Spearman rhos were used to determine the reliability of self-reported PAL with the MET values calculated and overall compliance to exercise prescription. The demographic groups in Table 1 had to be recoded into binomial categories for more accurate analysis. These categories include (a) sex (male vs. female); (b) age (youth: 18–39 years old versus adults: 40–65 years old); (c) physical attributes (disability vs. non-disabled); (d) state (Klang Valley vs. others); (e) education level (non-graduates versus graduates); (f) income group (B40 versus M40 and T20); (g) employment, education, or training status (yes vs. no); (h) type of mobility aid used (with or without aids); (i) partnership (no partner vs. partnered); and (j) race (Malays vs. non-Malays). The income range for the B40 group was valued at RM ≤ 4999, and the M40/T20 group had an income of RM ≥ 5000. This categorization used the guidelines reported by the Malaysian Department of Statistics [26].

The analysis for ADL for each PA used dimension reductions based on univariate descriptives with varimax orthogonal rotations. Criteria for factor loadings were based on an eigenvalue ≥1 and factor loading of ≥0.4. In addition, principal component dimension reduction and extraction were also conducted in combined and separate categories (non-disabled versus physical disability).

Participation in exercise was deemed achieved if individuals performed ≥2.56 MET h/week of moderate–vigorous exercises and at least ≥1.76 MET h/week of endurance training. This calculation was based on the PA guidelines recommended by health organizations [18]. Aerobic exercise is based on the accumulation of either item 4 (moderate exercise) or 5 (vigorous exercise) of the PASIPD questionnaire, having performed at least 1–2 days (seldom) per week of moderate-intensity (4 MET) exercise for 2–4 h per day [1]. Endurance training was based on performing at least 1–2 days (seldom) per week of resistance training for 1–2 h per day [1]. All tests used a *p* < 0.05 significance level.

## 3. Results

Table 1 describes the demographic profile of the surveyed respondents. A total of N = 176 non-disabled and 176 individuals with a physical disability were surveyed. Of the one hundred seventy-six individuals with physical disability, eighty-five had spinal cord injury, seventy-three had spine deformities, nine had spina bifida, two had cerebral palsy, and seven had other types of physical disability.

The MET values for each group of physical disability were valued at (in median, interquartile range): spinal cord injury (21.13, 32.14); CP (26.29, no IQR); spina bifida (16.26, 27.33); spine deformities (12.01, 13.32); stroke (12.78, no IQR); and others (13.30, 27.30).

### 3.1. Factor Component Dimension Extraction

The dimension reduction extracted for the non-disabled adult group consisted of four dynamic factors associated with ADL. The PA dimensions extracted in Table 2 were Factor 1: home maintenance (home repair and lawn and gardening work); Factor 2: household chores (light and heavy housework and caring for another person/pet/animal); Factor 3: exercise and training (moderate and vigorous exercise and endurance training); and Factor 4: light activities (leisure activities, light exercise, and occupational/volunteerism).

Within the physical disability group, there were four dimensions extracted (Table 3), consisting of Factor 1: leisure activities (leisure activities and recreation); Factor 2: home maintenance (home repair and lawn and gardening work); Factor 3: household chores (light and heavy housework and caring for another person/pet/animal); and Factor 4: career (occupational and volunteerism). Similarly, the combined group (Table 4) showed PA dimension reduction with four dynamic factors mirroring the physical disability group.

### 3.2. Physical Literacy and Physical Activity Level Reliability

Self-reported PAL, according to Jurca et al.’s five levels of PA score [23], correlated moderately well with their total MET h/day (*p* < 0.000, r = 0.57). Table 5 compares the PA performed by both groups. Although all of the respondents were aware of the health benefits of regular exercise, only 10 (2.8%) of the 356 individuals surveyed were able to correctly outline the exercise prescription guidelines as recommended by accredited global health agencies. A majority of those (eight out of ten) who answered correctly came from a career background related to sports and exercise. Twenty of the respondents (5.6%) were in possession of gym equipment that can produce moderate–vigorous exercise intensities, such as stationary bikes, treadmills, and exergaming machines. However, only a fifth of them (N = 4) actually reported using the equipment regularly (defined as 2–3 times a week ≥30 min).

### 3.3. Aerobic Exercise and Endurance Training Compliance

Only a handful (N = 45, 12.8%) of the participants surveyed complied with the minimum requirement set forth by health guidelines regarding aerobic exercise and resistance training prescriptions. Endurance training (N = 112, 31.8%) showed a higher compliance rate among the participants compared to aerobic exercise (N = 71, 20.2%) alone. There were significant negative correlations between self-reported PAL status and compliance to aerobic exercise, resistance training, and overall exercise prescriptions (*p* < 0.000, r = −0.24–0.29). Table 6 outlines the attributes and significance affecting compliance to exercise recommendations.

## 4. Discussion

This cross-sectional study on assessing the PL and comparing ADL between two groups of individuals (with or without physical disability) revealed surprising results. As opposed to a similar study comparing Malaysian children’s (with or without physical disability) PAL [27], this study showed that there was no significant difference in PAL between the non-disabled and physical disability groups. Even more surprising is the fact that there was a significantly higher number of achievers according to the recommended exercise prescription among those with physical disability, non-graduates, those from the lower income group (B40), and rural dwellers (outside of the highly dense Klang Valley area). These reports seem to be in tandem with other studies focusing on the Malaysian population, which indicate that lower income groups and those with lower education level had higher PAL values and exercise compliance [10,28,29,30]. In addition, PA patterns extracted from the factor analyses seem to point out the fact that ADL was mostly similar for home maintenance and household chores between the groups with and without physical disability. This indicated that both groups had a high degree of autonomy in managing their daily tasks and were able to survive independently despite one group having a form of physical disability. Physical independence is a very strong indicator for higher quality of life, as well as greater earning potential for the respondents [4,31].

However, PA differs greatly in terms of factors related to leisure activities, exercise, and training, as well as occupation. The factor analysis suggested that for the physical disability group, leisure, exercise, and training were a major part of their ADL, indicating those activities are part of leisure time physical activity (LTPA) performed outside of their normal commitments to work and living. Instead, work-related activities can be seen as optional obligations that take up the least time in their overall ADL. It could indicate that the physical disability group received financial benefits or were dependent on family support regarding finances. This identification is an important clue to explaining why exercise participation rates are much higher in this group. Indeed, it appears that they may have had more time at hand to partake in LTPA due to lower time consumed by career work, which also explains the link between lower income and higher exercise compliance.

In contrast, the non-disabled group relegated leisure, light exercise, and work requiring physical activities to a minor part of their total PAL. Additionally, moderate–vigorous exercise and endurance training came only after their commitments to home maintenance and household chores and were categorized under “exercise and training”. This segregation supports the notion that for the non-disabled group, exercise is part of a special ADL routine specifically for physical performance and enhancements, where light exercise is negligible. In addition, work obligations, which may have taken up a major part of their ADL, evident in their income bracket, were mostly of low PAL, suggesting that they might have indeed been blue-collar workers confined to their desk jobs.

The results of this survey indicated two very important aspects that researchers might have missed when dealing with sedentarism. The first is that the lifestyle pressures apparent among high income earners and graduates, especially those living in populated urban areas, may have affected their ability to participate in exercise regularly. This is more likely due to time constraints and insurmountable commitments to career and family that may have impacted their exercise habits [32]. Secondly, it is possible that city dwellers (who make up the majority of the high-income earners, graduates, and urban dwellers) are more likely to prioritize other forms of entertainment as part of their LTPA rather than participate in dose-potent exercise routines [33]. This may include less physically grueling LTPA such as going out to see movies, shopping, dining at restaurants, or attending musical events.

In addition to this, there was also no significant difference in exercise compliance between sex in the study. Such a finding indicated either of two reasons; one is that they both view exercise as a lower priority compared to other forms of ADL or LTPA due to poor motivation [34]. The other reason could potentially be that both sexes found it equally impossible to find time to perform health-beneficial exercises due to their massive commitments to daily responsibilities [35]. It could be in the form of commitments to household chores, home maintenance, or work-related priorities. This was evident from the very low consistency of exercise equipment used even at home. At this stage, with the study design, it is difficult to ascertain whether there are any biases against or aversions toward female participation in specific physical activities. The situation points toward two important directions in term of promoting exercise for Malaysians. The first is that exercise programs should be unisex in nature, targeting both sexes and enhancing mixed-gender sports for greater participation. The second objective should be to design exercises that are time-, cost-, and space-efficient enough to entice busy working adults to participate. The introduction of e-sports exergaming may be of interest as it allows portability, can be practiced at home or anywhere around the world, and is space-efficient enough to double as entertainment in the living room [36,37,38].

However, the overall report has shown that although exercise prescription compliance was low (12.8%), both groups were generally active, being within the lower end of the moderately active spectrum (defined as between 11–50 MET h/day) as outlined by WHO [4]. In context, the minimum energy expenditure recommended by health guidelines to provide protective measures against cardiovascular heart disease is at least 10 MET h/week [1,39,40]. On average, non-disabled respondents had lower PAL values compared to their counterparts with physical disability, clocking a MET value of 12.77 (21.44) h per day versus 14.30 (19.76). The factor analysis and significant difference in MET values for ADL categories indicate that non-disabled individuals are more prone to sedentary forms of LTPA (leisure and light exercise) and stationary desk jobs compared to the group with physical disability. The average PAL reported among Malaysians was between 3.77–18.92 MET h/day [16,27,28]. This may highlight the fact that individuals with physical disability were more likely to have wider opportunities or free time at hand to participate in LTPA involving leisure, light exercise, and physical forms of work, since more of them did not participate in employment, education, or training. As opposed to Malaysian children with physical disability, who exhibit extremely sedentary behavior [27], adults with physical disability demonstrate higher PL, PAL, and ability to survive independently based on the factors in ADL assessed in this survey.

Although the results indicate a more positive trend toward PAL overall, exercise habits as recommended by international health guidelines were still considerably low (12.8%). This is coupled with the very low PL on prescribed exercise recommendations set forth by international health guidelines (2.8%). In fact, all the respondents who were aware of the exercise recommendations were in tertiary education, suggesting that general health knowledge is linked to higher levels of education. In response to this report, it is imperative that more effort must be exerted to both enlighten the general public on recommended exercise regiments and educate them on appropriate exercises based on intensity, duration, endurance, and frequency performed.

Another surprising finding in this study is that even though PL is higher among the non-disabled, compliance to exercise prescription was higher in those with physical disability (or requiring mobility aids). In contrast to reported PA habits in developed countries, however, Malaysians with higher education levels were less likely to engage in health-beneficial exercise [11,41]. This can be due to the non-disabled group being more occupied with career work, which reduced the amount of time they can allocate for health beneficial LTPA. The findings suggest that there is an inherent difference in priorities involving LTPA among the non-disabled and those with a physical disability. As reported earlier, it is more likely that non-disabled individuals were prone to participating in LTPAs that are less intensive or grueling in nature as a form of destressing mechanism [32], since they are expected to perform well in their careers and earn more for the household. This is because high-intensity exercise has been linked to pain and discomfort, a negative emotion that is averse to engagement in exercise [42]. Therefore, future exercise programs should be more enjoyable and less intensive in nature. Although regular intervals of high-intensity exercise have been shown to increase mental health resilience, such laborious forms of LTPA seem to be unpopular among the tertiary educated, high income earners of the group. Indeed, due to this, health practitioners need new techniques [43] for promoting exercise to entice tertiary educated, high income earners to newer forms of training programs that are suited to their needs. Without the added intervention within this group, the highly sought-after goal of reducing up to 15% of sedentarism among the population [4] would not be achieved.

Additionally, the study reported moderately good correlations (r = 0.57) between self-reported PAL status [23] and total MET values per day. The correlation highlights that respondents are generally self-aware of their PAL status, which provides the needed insight to improve physical performance. This is also a good indicator to measure values that can be useful in future studies to assess physical performance or improvements in aerobic capacity based on questionnaires. Further research is needed to understand the in-depth reasons for low PAL or non-compliance to exercise, whether it is associated with barriers to exercise, facilitators of continued participation in health-beneficial LTPA, or encouragement of more independence in ADL. The results of this study can help pave new areas in linking fundamental issues between low exercise compliance against demographic factors.

## 5. Limitations

One limitation to this study can be the fact that the findings may only cover the tip-of-the-iceberg group of individuals who were accessible and willing to participate in this study. This may have resulted in higher levels of reported PAL, as they are likely to be more active than the unreachable groups of people that were difficult to contact. These groups can include those with mental health issues, extremely low economic backgrounds, and living in very rural communities. This can result in higher PAL than the expected value since individuals with contraindications to moderate–vigorous exercise was also excluded from this survey.

Secondly, as with all questionnaire-based study designs, reporting or recall bias can affect the results by showing PAL trends instead of the actual ADL performed. The items listed in the PASIPD showed acceptable to good reliability (α = 0.54–0.75), with acceptable factor-based intraclass correlations (ICC = 0.12–0.53) for both the groups sampled. Additionally, the moderately good correlation between the self-reported PAL and MET values (r = 0.57) indicate that the results are acceptable mediums for study analysis. The PASIPD questionnaire has been reported to provide low-to-good internal consistency (ICC = 0.20–0.87), poor-to-moderate correlation with PAL (r = 0.22–0.51), and comparisons with wearable devices such as accelerometers [16,44,45,46,47,48,49,50]. However, such poor-to-moderate measures of internal consistency and correlations are also apparent in other forms of PA questionnaires, such as the well-known International Physical Activity Questionnaire (IPAQ) [51]. Given that the study is large-scale in nature, with 352 respondents surveyed, the use of a questionnaire is deemed more appropriate as it is more homogenous and standardized internationally due to its reliance on the MET for energy expenditure reporting, for individuals both with and without physical disability. Because of these results, it is also advised to interpret the results with caution and take into consideration the reported low consistencies and poor correlations.

Finally, due to the study design being cross-sectional in nature, determination of any causal relationship between low PAL or non-compliance to moderate–vigorous exercise participation with socioeconomic or demographic barriers was not possible. The correlations between these various factors may assist in further understanding the underlying reasons behind low PAL or low compliance to exercise. This will help in developing better exercise programs that can improve compliance or attract more adherence from targeted individuals.

## 6. Conclusions

The cross-sectional study, comparing PAL and ADL between non-disabled and adults with physical disability, showed that the overall MET h per day performed indicate moderately active PAL for both groups (12.77–14.30 MET h/day). The group with physical disability reported higher median PAL values as opposed to the non-disabled group, with significant differences seen in light exercise, endurance training, housework, and occupational work. However, the overall compliance to exercise as prescribed by international health guidelines was still low at 12.8%. The study also highlights a very low PL (2.8%) in health recommended exercise guidelines.

## Figures and Tables

**Table 1:** Respondents’ demography, N = 352.

Variable	Non-Disabled	Physical Disability	Combined
N, sample size	176	176	352
Sex	Male: 85 Female: 91	Male: 101 Female: 75	Male: 186 Female: 166
* Age	27 (14)	33 (15)	30 (14)
Race	Malay: 92 Chinese: 73 Others: 11	Malay: 89 Chinese: 77 Others: 10	Malay: 181 Chinese: 150 Others: 21
State	Klang Valley: 145 Other states: 31	Klang Valley: 103 Other states: 73	Klang Valley: 248 Other states: 104
Education level	Non-graduates: 32 Graduates: 144	Non-graduates: 90 Graduates: 86	Non-graduates: 122 Graduates: 230
EET status	Yes: 172 No: 4	Yes: 126 No: 50	Yes: 298 No: 54
Partnership	With partner: 90 No partner: 86	With partner: 96 No partner: 80	With partner: 186 No partner: 166
Income group	B40: 83 M40 and T20: 93	B40: 95 M40 and T20: 81	B40: 178 M40 and T20: 174
Mobility aid	Walking aids: 0 No aid: 176	Walking aids: 107 No aid: 69	Walking aids: 108 No aid: 244
* PAL (MET h/day)	Male: 12.83 (20.2) Female: 12.67 (22.92)	Male: 15.29 (24.38) Female: 13.44 (17.99)	Male: 12.8 (20.2) Female: 12.7 (22.9)

* Values reported in median and interquartile range. Abbreviations: MET = metabolic equivalent of a task; PAL = physical activity level; EET = employment, education, or training.

**Table 2:** Factor analysis of physical activities in adults (non-disabled).

Activity	α if Item Deleted	Factor 1 (Home Maintenance)	Factor 2 (Household Chores)	Factor 3 (Exercise and Training)	Factor 4 (Light Activities)
Leisure activities	0.56				0.70
Light exercise	0.57				0.43
Moderate exercise	0.56			0.48	
Vigorous exercise	0.54			0.85	
Endurance training	0.54			0.86	
Light housework	0.58		0.81		
Heavy housework	0.55		0.75		
Home repair	0.58	0.56			
Lawn work	0.57	0.87			
Gardening	0.57	0.90			
Caring for person/pet	0.57		0.76		
Occupational/ volunteerism	0.65				0.70
Cronbach’s α	0.72 (overall)	0.78	0.74	0.69	0.378
Intraclass correlation	0.11 (overall)	0.53	0.46	0.401	0.118
Eigenvalue		2.30	2.07	1.90	1.42
Variance (%)		19.12	17.23	15.82	11.81
Cumulative variance (%)		19.12	36.36	52.18	63.99

**Table 3:** Factor analysis of physical activities in adults (physical disability).

Activity	α if Item Deleted	Factor 1 (Leisure Activities)	Factor 2 (Home Maintenance)	Factor 3 (Household Chores)	Factor 4 (Career)
Leisure activities	0.68	0.67			
Light exercise	0.68	0.74			
Moderate exercise	0.65	0.82			
Vigorous exercise	0.69	0.74			
Endurance training	0.69	0.63			
Light housework	0.70			0.82	
Heavy housework	0.69			0.74	
Home repair	0.71		0.65		
Lawn work	0.72		0.93		
Gardening	0.72		0.93		
Caring for person/pet	0.72			0.78	
Occupational/ volunteerism	0.71				0.86
Cronbach’s α	0.75 (overall)	0.78	0.80	0.72	NA
Intraclass correlation	0.17 (overall)	0.35	0.51	0.44	NA
Eigenvalue		2.96	2.20	1.94	1.05
Variance (%)		24.69	18.36	16.13	8.74
Cumulative variance (%)		24.69	43.04	59.18	67.92

**Table 4:** Factor analysis of physical activities in adults (combined).

Activity	α if Item Deleted	Factor 1 (Leisure Activities)	Factor 2 (Home Maintenance)	Factor 3 (Household Chores)	Factor 4 (Career)
Leisure activities	0.69	0.58			
Light exercise	0.69	0.67			
Moderate exercise	0.68	0.80			
Vigorous exercise	0.69	0.75			
Endurance training	0.68	0.68			
Light housework	0.70			0.82	
Heavy housework	0.69			0.79	
Home repair	0.70		0.61		
Lawn work	0.71		0.92		
Gardening	0.71		0.93		
Caring for person/pet	0.71			0.75	
Occupational/volunteerism	0.69				0.89
Cronbach’s α	0.80 (overall)	0.75	0.80	0.73	NA
Intraclass correlation	0.16 (overall)	0.33	0.52	0.45	NA
Eigenvalue		2.60	2.17	1.97	1.10
Variance (%)		21.68	18.05	16.45	9.18
Cumulativevariance (%)		21.68	39.72	56.17	65.36

**Table 5:** Physical activities performed by individuals with and without physical disabilities.

Activities (in MET h/Day)	Non-Disabled, (N = 176)	Physical Disability, (N = 176)	*p* Value	95% CI	Effect Size (Cliff’s d)	Combined (N = 352)
Leisure activities	1.60 (3.13)	1.88 (5.63)	0.257	0.134–0.148	0.054	1.88 (3.13)
Light exercise	0.33 (0.96)	0.96 (2.25)	* 0.001	0.000–0.0010	0.10	0.33 (1.29)
Moderate exercise	0.00 (1.28)	0.00 (1.61)	0.257	0.085–0.097	0.054	0.00 (1.28)
Vigorous exercise	0.00 (2.00)	0.00 (2.56)	0.545	0.19–0.20	0.043	0.00 (2.56)
Endurance training	0.61 (1.38)	0.61 (3.62)	* 0.032	0.0080–0.011	0.077	0.61 (2.37)
Light housework	0.65 (1.56)	0.48 (1.77)	* 0.012	0.0030–0.0050	0.085	0.65 (1.77)
Heavy housework	0.44 (1.28)	0.35 (1.28)	* 0.002	0.0000–0.0010	0.054	0.44 (1.28)
Home repair	0.00 (0.00)	0.00 (0.00)	0.808	0.15–00.17	0.034	0.00 (0.00)
Lawn work	0.00 (0.00)	0.00 (0.00)	0.999	0.51–0.53	0.020	0.00 (0.00)
Gardening	0.00 (0.44)	0.00 (0.00)	1.000	0.72–0.74	0.017	0.00 (0.00)
Caring for person/pet	0.00 (1.61)	0.00 (1.85)	1.000	0.98–0.98	0.014	0.00 (1.61))
Occupational/ volunteerism	0.00 (7.78)	0.00 (1.60)	* 0.023	0.0030–0.0060	0.080	0.00 (3.75)
Total MET Mean (SD)	18.00 (15.70) 12.77 (21.44)	20.97 (22.85) 14.30 (19.76)	0.723	0.72–0.73	0.039	19.48 (19.63) 13.49 (20.75)

* Significant difference at the level of *p* < 0.05. Values reported in median and interquartile range unless otherwise stated. Abbreviations: MET = metabolic equivalent of a task; CI = confidence interval.

**Table 6:** Comparison of exercise compliance based on different categorical attributes.

Variable	Complied (N)	Not Complied (N)	*p* Value
Sex	Male: 31 Female: 14	Male: 155 Female: 152	0.140
Physical attributes	Non-disabled: 0 Physical disability: 45	Non-disabled: 176 Physical disability: 131	* 0.000
State of residence	Klang Valley: 18 Other states: 27	Klang Valley: 230 Other states: 77	* 0.000
Race	Malays: 30 Non-Malays: 15	Malays: 151 Non-Malays: 156	0.182
Partnership	With partner: 21 No partner: 24	With partner: 165 No partner: 142	0.989
Education level	Non-graduates: 34 Graduates: 11	Non-graduates: 88 Graduates: 219	* 0.000
Employment, education, or training (EET) status	Yes: 31 No: 14	Yes: 267 No: 40	0.154
Income group	B40: 34 M40 and T20: 11	B40: 144 M40 and T20: 163	* 0.003
Mobility aid	Walking aids: 36 No aid: 9	Walking aids: 72 No aid: 235	* 0.000

* Significant difference at the level of *p* < 0.05.

## Data Availability

Data is not available due to privacy and ethical restrictions.

## References

[1] Garber C.E., Blissmer B., Deschenes M.R., Franklin B.A., Lamonte M.J., Lee I.M., Nieman D.C., Swain D.P., American College of Sports Medicine (2011). American College of Sports Medicine position stand. Quantity and quality of exercise for developing and maintaining cardiorespiratory, musculoskeletal, and neuromotor fitness in apparently healthy adults: Guidance for prescribing exercise. Med. Sci. Sports Exerc..

[2] World Health Organization (2000). Obesity: Preventing and Managing the Global Epidemic.

[3] World Health Organization (2010). Global Recommendations on Physical Activity for Health.

[4] World Health Organization WHO Guidelines on Physical Activity and Sedentary Behaviour: At a Glance. https://www.who.int/publications/i/item/9789240014886.

[5] World Health Organization (2022). Physical Inactivity a Leading Cause of Disease and Disability, Warns WHO.

[6] Whitehead M. (2001). The concept of physical literacy. Eur. J. Phys. Educ..

[7] Whitehead M. (2010). Physical Literacy: Throughout the Lifecourse.

[8] (2017). The International Physical Literacy Association. https://www.physical-literacy.org.uk/.

[9] Tremblay M.S., Costas-Bradstreet C., Barnes J.D., Bartlett B., Dampier D., Lalonde C., Leidl R., Longmuir P., McKee M., Patton R. (2018). Canada’s physical literacy consensus statement: Process and outcome. BMC Public Health.

[10] Cai Lian T., Bonn G., Si Han Y., Chin Choo Y., Chee Piau W. (2016). Physical activity and its correlates among adults in Malaysia: A cross-sectional descriptive study. PLoS ONE.

[11] Bauman A.E., Sallis J.F., Dzewaltowski D.A., Owen N. (2002). Toward a better understanding of the influences on physical activity: The role of determinants, correlates, causal variables, mediators, moderators, and confounders. Am. J. Prev. Med..

[12] Ginis K., Latimer A., Hicks A., Craven C. (2005). Development and evaluation of an activity measure for people with spinal cord injury. Med. Sci. Sports Exerc..

[13] Mat Rosly M., Halaki M., Hasnan N., Mat Rosly H., Davis G.M., Husain R. (2018). Leisure time physical activity participation in individuals with spinal cord injury in Malaysia: Barriers to exercise. Spinal Cord.

[14] Vissers M., van den Berg-Emons R., Sluis T., Bergen M., Stam H., Bussmann H. (2008). Barriers to and facilitators of everyday physical activity in persons with a spinal cord injury after discharge from the rehabilitation centre. J. Rehabil. Med..

[15] Guthold R., Stevens G.A., Riley L.M., Bull F.C. (2020). Global trends in insufficient physical activity among adolescents: A pooled analysis of 298 population-based surveys with 1.6 million participants. Lancet Child Adolesc. Health.

[16] Mat Rosly M., Halaki M., Mat Rosly H., Davis G.M., Hasnan N., Husain R. (2020). Malaysian adaptation of the physical activity scale for individuals with physical disabilities in individuals with spinal cord injury. Disabil. Rehabil..

[17] Hills A.P., Mokhtar N., Byrne N.M. (2014). Assessment of physical activity and energy expenditure: An overview of objective measures. Front. Nutr..

[18] Washburn R.A., Zhu W., McAuley E., Frogley M., Figoni S.F. (2002). The physical activity scale for individuals with physical disabilities: Development and evaluation. Arch. Phys. Med. Rehabil..

[19] Beaton D.E., Bombardier C., Guillemin F., Ferraz M.B. (2000). Guidelines for the process of cross-cultural adaptation of self-report measures. Spine.

[20] Sousa V.D., Rojjanasrirat W. (2011). Translation, adaptation and validation of instruments or scales for use in cross-cultural health care research: A clear and user-friendly guideline. J. Eval. Clin. Pract..

[21] Balady G.J., Chaitman B., Driscoll D., Foster C., Froelicher E., Gordon N., Pate R., Rippe J., Bazzarre T. (1998). AHA/ACSM joint position statement: Recommendations for cardiovascular screening, staffing, and emergency policies at health/fitness facilities. Med. Sci. Sports Exerc..

[22] Ritter P., Lorig K., Laurent D., Matthews K. (2004). Internet versus mailed questionnaires: A randomized comparison. J. Med. Internet Res..

[23] Jurca R., Jackson A.S., LaMonte M.J., Morrow J.R.J., Blair S.N., Wareham N.J., Haskell W.L., van Mechelen W., Church T.S., Jakicic J.M. (2005). Assessing cardiorespiratory fitness without performing exercise testing. Am. J. Prev. Med..

[24] Wilson VanVoorhis C.R., Morgan B.L. (2007). Understanding Power and Rules of Thumb for Determining Sample Sizes. Tutor. Quant. Methods Psychol..

[25] Macbeth G., Razumiejczyk E., Ledesma R.D. (2011). Cliff’s Delta Calculator: A non-parametric effect size program for two groups of observations. Univ. Psychol..

[26] Department of Statistics Malaysia (2019). Household Income and Basic Amenities Survey Report. https://www.dosm.gov.my/v1/index.php?r=column/cthemeByCat&cat=120&bul_id=TU00TmRhQ1N5TUxHVWN0T2VjbXJYZz09&menu_id=amVoWU54UTl0a21NWmdhMjFMMWcyZz09.

[27] Mat Rosly M. (2022). Physical activity and exercise participation among Malaysian children (able-bodied vs. physical disability): A cross-sectional study. Children.

[28] Nik-Nasir N.M., Md-Yasin M., Ariffin F., Mat-Nasir N., Miskan M., Abu-Bakar N., Yusoff K., REDISCOVER Investigators (2022). Physical activity in Malaysia: Are we doing enough? Findings from the REDISCOVER study. Int. J. Environ. Res. Public Health.

[29] Tam C.L., Bonn G., Yeoh S.H., Wong C.P. (2014). Investigating diet and physical activity in Malaysia: Education and family history of diabetes relate to lower levels of physical activity. Front. Psychol..

[30] Pell C., Allotey P., Evans N., Hardon A., Imelda J.D., Soyiri I., Reidpath D.D., SEACO Team (2016). Coming of age, becoming obese: A cross-sectional analysis of obesity among adolescents and young adults in Malaysia. BMC Public Health.

[31] Ramakrishnan K., Loh S.Y., Omar Z. (2011). Earnings among people with spinal cord injury. Spinal Cord.

[32] Suhaimi S.A., Müller A.M., Hafiz E., Khoo S. (2022). Occupational sitting time, its determinants and intervention strategies in Malaysian office workers: A mixed-methods study. Health Promot. Int..

[33] Liu K.T., Kueh Y.C., Arifin W.N., Ismail Ibrahim M., Shafei M.N., Kuan G. (2020). Psychometric properties of the decisional balance scale: A confirmatory study on Malaysian university students. Int. J. Environ. Res. Public Health.

[34] Molanorouzi K., Khoo S., Morris T. (2015). Motives for adult participation in physical activity: Type of activity, age, and gender. BMC Public Health.

[35] Justine M., Azizan A., Hassan V., Salleh Z., Manaf H. (2013). Barriers to participation in physical activity and exercise among middle-aged and elderly individuals. Singap. Med. J..

[36] Mat Rosly M., Mat Rosly H. (2023). Home-based exergaming training effects for two individuals with spinal cord injury: A case report. Physiother. Theory Pract..

[37] Tripette J., Murakami H., Gando Y., Kawakami R., Sasaki A., Hanawa S., Hirosako A., Miyachi M. (2014). Home-based active video games to promote weight loss during the postpartum period. Med. Sci. Sports Exerc..

[38] Mat Rosly M., Mat Rosly H., Hasnan N., Davis G.M., Husain R. (2017). Exergaming boxing versus heavy bag boxing: Are these equipotent for individuals with spinal cord injury?. Eur. J. Phys. Rehabil. Med..

[39] Tanasescu M., Leitzmann M.F., Rimm E.B., Willett W.C., Stampfer M.J., Hu F.B. (2002). Exercise type and intensity in relation to coronary heart disease in men. JAMA.

[40] Lee I.M., Rexrode K.M., Cook N.R., Manson J.E., Buring J.E. (2001). Physical activity and coronary heart disease in women: Is “no pain, no gain” passe?. JAMA.

[41] Hawkins M.S., Storti K.L., Richardson C.R., King W.C., Strath S.J., Holleman R.G., Kriska A.M. (2009). Objectively measured physical activity of USA adults by sex, age, and racial/ethnic groups: A cross-sectional study. Int. J. Behav. Nutr. Phys. Act..

[42] Smith P.J., Merwin R.M. (2021). The role of exercise in management of mental health disorders: An integrative review. Annu. Rev. Med..

[43] Müller A.M., Khoo S., Morris T. (2016). Text messaging for exercise promotion in older adults from an upper-middle-income country: Randomized controlled trial. J. Med. Internet Res..

[44] Van der Ploeg H.P., Streppel K.R., van der Beek A.J., van der Woude L.H., Vollenbroek-Hutten M., van Mechelen W. (2007). The Physical Activity Scale for Individuals with Physical Disabilities: Test-retest reliability and comparison with an accelerometer. J. Phys. Act. Health.

[45] Van den Berg-Emons R.J., L’Ortye A.A., Buffart L.M., Nieuwenhuijsen C., Nooijen C.F., Bergen M.P., Stam H.J., Bussmann J.B. (2011). Validation of the Physical Activity Scale for Individuals with Physical Disabilities. Arch. Phys. Med. Rehabil..

[46] De Groot S., van der Woude L.V.H., Niezen A., Smit C.A., Post M.W. (2010). Evaluation of the physical activity scale for individuals with physical disabilities in people with spinal cord injury. Spinal Cord.

[47] Meunier P., Joussain C., Gremeaux V., Carnet D., Bastable P., Ruet A., Drigny J. (2021). Transcultural adaptation and validation of a French version of the Physical Activity Scale for Individuals with Physical Disabilities (PASIPD-Fr). Ann. Phys. Rehabil. Med..

[48] Jimenez-Pardo J., Holmes J.D., Jenkins M.E., Johnson A.M. (2015). An examination of the reliability and factor structure of the Physical Activity Scale for Individuals with Physical Disabilities (PASIPD) among individuals living with Parkinson’s Disease. J. Aging Phys. Act..

[49] Ulaş K., Topuz S., Horasan G. (2019). The validity and reliability of the Turkish version of the Physical Activity Scale for Individuals with Physical Disabilities (PASIPD). Turk. J. Med. Sci..

[50] Golin A., Costa E.C., Assis I.S.A., Makhoul M.P., Barbieri F.A., Torriani-Pasin C. (2022). Translation, cross-cultural adaptation, and validation of the Physical Activity Scale for Individuals with Physical Disabilities for Brazilian individuals with Parkinson’s Disease. J. Aging Phys. Act..

[51] Craig C.L., Marshall A.L., Sjöström M., Bauman A.E., Booth M.L., Ainsworth B.E., Pratt M., Ekelund U., Yngve A., Salli J.F. (2003). International physical activity questionnaire: 12-country reliability and validity. Med. Sci. Sports Exerc..

